# Factors Associated with Provider Practices Related to Infant Feeding in Primary Care Settings: Results from a Pilot Survey

**DOI:** 10.3390/nu16020179

**Published:** 2024-01-05

**Authors:** Hope K. Lima, Meghan Ganio Molinari, Jessie B. Hoffman, Lisa Akers, Karin I. Evans, Ashley Licata

**Affiliations:** 1 Department of Human Nutrition, Winthrop University, Rock Hill, SC 29733, USA; msmolinari@novanthealth.org (M.G.M.); hoffmanjb@winthrop.edu (J.B.H.); evansk@winthrop.edu (K.I.E.); ashley.licata@samford.edu (A.L.); 2 Novant Health Presbyterian Medical Center, Charlotte, NC 28204, USA; 3 Gretchen Swanson Center for Nutrition, Omaha, NE 68154, USA; lakers@centerfornutrition.org; 4 School of Public Health, Samford University, Homewood, AL 35229, USA

**Keywords:** lactation, primary care, survey validation, coordinated care

## Abstract

In 2020, only 25.6% of dyads in the US were exclusively breastfeeding at six months. Previous research has shown that breastfeeding continuation improves when patients receive both prenatal and postpartum support. Additionally, breastfeeding self-efficacy can be directly impacted by interactions with primary healthcare providers. To facilitate improved lactation support and positive interactions with providers related to infant feeding in the primary care setting, a 49-question survey was utilized to conduct a retrospective, cross-sectional study. Using multiple regression analysis, the researchers tested a model to determine if certain factors could predict patients receiving lactation education in the primary care setting. The full model was statistically significant and accounts for 81.8% of the variance (R^2^ = 0.818, F (7, 21) = 9.015, *p* < 0.001, CI = 0.728 to 0.910). Variables that contributed significantly to the model included provider age, provider years of experience in maternal-child health, population density of the practice, and average provider preparedness and comfort with lactation support and medical management. As the only modifiable predictor significantly contributing to the model, future research is necessary to develop educational interventions to improve provider preparedness and comfort with lactation support and medical management. Such interventions may significantly improve the frequency of lactation education in primary care settings.

## 1. Introduction

Human milk is the ideal source of nutrition for infants due to the physical, social, and cognitive benefits it imparts on maternal-infant health [1,2,3]. Efforts such as the Baby Friendly Hospital Initiative^®^ (BFHI) have increased breastfeeding initiation rates in the United States from 73.8% in 2004 to 84.1% in 2020 [4,5]. Despite these gains, however, only 24.9% of infants were exclusively breastfed to six months in the United States in 2019 [5]. The steep drop off between initiation and exclusivity at six months highlights the need for additional support after discharge from the hospital to ensure that parents are able to continue to feed their children human milk.

Breastfeeding self-efficacy, the belief that one is physically able to carry out the process of lactation and will successfully feed their infant, is one modifiable determinant of duration and exclusivity [6]. Studies support the finding that breastfeeding self-efficacy is impacted by interactions with healthcare practitioners [7,8]. Moreover, breastfeeding outcomes improve when women and families receive a combination of prenatal education coupled with postnatal support [9,10]. Primary care providers in outpatient settings have a unique opportunity to promote and encourage human milk as an extension of their positions. Unfortunately, several determinants exist which may negatively impact breastfeeding support in primary care [11,12,13].

Of specific interest, current literature indicates that lactation curriculum in medical residency programs is insufficient [14,15,16,17,18]. Pediatric residents have been reported to receive an average of nine hours of breastfeeding education over three years, while many family physician residents report receiving less than four hours [16,18]. Additionally, the majority (~80%) of well-child visits have been reported to last less than 20 min [19]. Data on postpartum OB/GYN care show average postpartum visit length in the US is also less than 20 min [20] and only ~50% of women attend their 6-week postpartum visit with their primary care provider [21,22]. These data highlight provider knowledge and the amount of time spent with patients as potential barriers in providing lactation education and support in primary care settings.

The role of midwives, nurses, and dietitians in providing lactation education and support in primary care settings cannot be understated. During both the prenatal and postpartum period, these healthcare providers can influence breastfeeding success [11,12,23,24]. It is possible, however, that the limitations experienced by physicians may also be experienced by ancillary providers [25,26]. Given the importance of these providers, assessing factors that influence whether or not they are providing this support to their patients is equally essential.

Current research focused on the primary care setting has explored provider attitudes and beliefs about breastfeeding and how health professionals are currently supporting breastfeeding [23,24,25,26]. However, to our knowledge, there is no research that investigates the relationship between primary care provider beliefs and practices and the frequency with which their patients receive lactation education. To facilitate interventions aimed at improving breastfeeding duration rates, we need an understanding of provider perceptions and practices related to infant feeding specifically in a primary care setting. Thus, our research aim was to describe the perceptions and practices of practitioners involved in primary care for pregnant, postpartum, and infant populations related to lactation education, support, and medical management. Additionally, we aimed to develop a multiple regression model that would facilitate prediction of factors associated with patients receiving lactation education.

## 2. Materials and Methods

### 2.1. Survey Development

Three domains and sub-dimensions of lactation education and support practices in primary care settings were determined based on need in the field and frequency in the literature: provider perceptions and roles, provider practices, and coordination of care across ancillary providers. Researchers generated items for all identified domains and associated sub-dimensions and organized them into a logical flow. Table A1 provides examples of sub-dimensions and associated questions within the three identified domains. The preliminary instrument was a 49-question survey entitled “Assessing Healthcare Provider Perceptions and Roles: A Survey on Lactation Practices in Primary Care Settings”.

### 2.2. Survey Content and Face Validation

Researchers recruited experts via email who were actively practicing in clinical primary care settings with pregnant and lactating populations to serve as content validators. A total of seven content validators provided expert feedback, which is above the minimum recommended for calculating a content validity index (CVI) [27]. In nutrition research, CVI is accepted as the standard of content validity practice [28]. In order to calculate the CVI, each content validator was asked to read and rate each survey question using a 4-point Likert scale from one (irrelevant) to four (extremely relevant) as it pertained to lactation care from the perspective of their practice. The content validators could also use a general comments box for open-ended feedback and any proposed revisions.

Researchers utilized the scoring guide previously published by Lynn [27] to create a score for each item. Per the tool guidelines, items receiving a CVI of less than 0.86 were removed. Items receiving a CVI of 0.86–0.99 were assessed and given minor alterations based on expert feedback prior to circulating the instrument to selected experts for face validation. Items with a CVI of 1.0 were kept in their original format. The CVI for the whole instrument was calculated by determining the proportion of total items judged to be content valid (CVI of >0.86). Two questions were removed from the survey due to receiving a CVI rating of <0.86. Based on total expert agreement, seven questions were added with the intent to clarify clinician practices and resources. The calculated CVI for the whole instrument was 0.95.

Specific feedback from four out of seven experts during the content validation stage indicated the need for definitions surrounding the terms used in the survey, specifically “lactation education”, “lactation support”, and “lactation medical management”, to ensure consistent feedback from survey participants. For the purposes of furtherance of research in this field, the researchers propose definitions for lactation education, lactation support, and lactation medical management, with the intent of clarifying care practices provided in different settings (Table A2).

After all content validation feedback was incorporated, the researchers recruited experts via email who conduct survey-based research and have knowledge of current clinical lactation practices in the United States. The panel was asked to read and provide qualitative feedback on the format of the survey, readability of survey questions, ease of use for respondents, and whether the survey included all necessary lactation concepts in the context of lactation practices in the United States. A total of three experts provided an assessment during the face validation process, which is within the appropriate range of experts recommended for face validation [27]. During the face validation stage, one demographic qualifier and three questions were added based on total expert agreement. After both content and face validation were complete, the survey length had increased by a total of nine questions (18.4% change).

### 2.3. Research Design

A retrospective, cross-sectional study design was used to assess primary care provider perceptions of lactation practices and the role of lactation professionals in outpatient, primary care settings. This survey was considered exempt by the Winthrop University Institutional Review Board. The final 58-question survey was loaded to Qualtrics Software (Provo, UT, USA, version is October 2023). Informed consent was provided at the initiation of the survey. Information obtained during the survey procedure was recorded in a manner such that respondents were not identifiable.

Participants were recruited using publicly available information, gathered via websites and social media platforms. The survey was distributed via electronic mail using non-probability convenience sampling from February 2020 through June 2020. The target population was defined as English-speaking providers with a primary care credential who were over the age of 18 and who provide care to pregnant, lactating, or infant populations in an outpatient setting. Distribution was focused in the Southeastern United States, including Alabama, Florida, Georgia, Kentucky, North Carolina, Tennessee, and South Carolina. A total of 132 unique responses were recorded with complete data on variables of interest.

### 2.4. Data Analysis

Data were coded and analyzed with IBM SPSS Statistics Version 29.0.0.0 (Armonk, NY, USA). Descriptive statistics and frequencies were run on variables of interest. Multiple regression analysis was used to test the hypothesis that age, whether or not the provider has an IBCLC, years of experience in maternal-child health, population density of the practice location, the frequency with which the provider initiates a conversation about breastfeeding, and the preparedness and comfort with providing lactation support and medical management would collectively influence the frequency with which patients in the practice receive lactation education. The assumptions of data normality, linearity, homoscedasticity, and absence of multicollinearity were checked prior to testing.

## 3. Results

**Figure 1:**
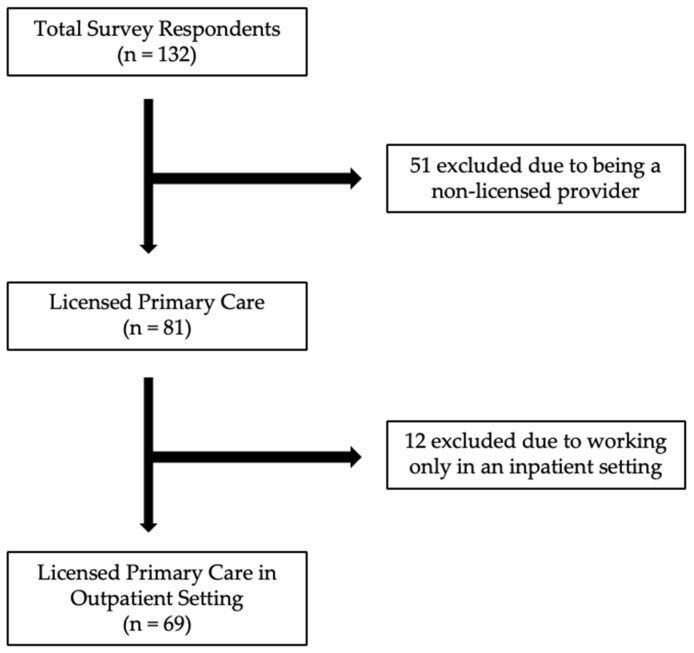
Study inclusion and exclusion flow diagram.

The survey was distributed to approximately 632 members across seven organizations. A total of *n* = 132 respondents completed the survey (a 21% response rate). Final data analysis was completed for *n* = 69 participants (Figure 1).

### 3.1. Characteristics of Respondents

The sample (*n* = 69) was largely composed of providers with a Medical Doctor (MD) or Doctor of Osteopathic Medicine (DO) credential (59.4%). Additionally, 20.3% of respondents reported concurrently holding the International Board Certified Lactation Consultant (IBCLC) credential. Average reported history of practice in maternal-child health was 15 years (SD = 13.2, range: 1–50). Further demographic and clinical characteristics are reported in Table 1.

### 3.2. Provider Perceptions and Roles

Of the respondents providing care during pregnancy (*n* = 31), 80.6% of providers indicate that patients request information about breastfeeding during pregnancy. Additionally, 64.0%, 52.0%, and 52.0% of providers report that patients request education about formula feeding, mixed feeding, and nutrition for lactation, respectively. Of the respondents providing care during the postpartum period to the parent or the child (*n* = 68), only 57.4% report receiving patient requests for information on breastfeeding. Only 38.2% and 35.3% of providers reported receiving requests for information about formula feeding or mixed feeding during the postpartum period, respectively. The data are summarized in Table 2.

**Figure 2:**
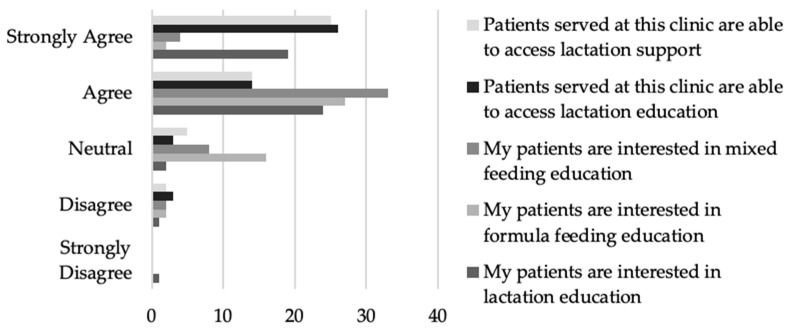
Provider perceptions related to accessibility and interest in infant feeding support and education.

Of the providers that responded, the majority agree or strongly agree that lactation support (*n* = 40, 84.7%) and lactation education (*n* = 41, 86.9%) are accessible (Figure 2).

**Figure 3:**
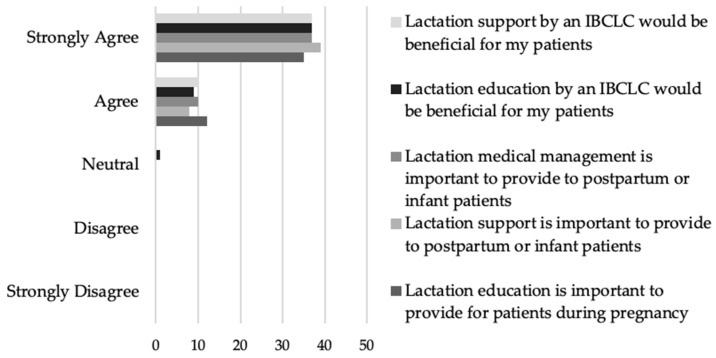
Provider beliefs about the importance and benefit of lactation education, support, and medical management.

Additionally, the providers who responded unanimously agreed or strongly agreed that lactation education (*n* = 47, 100%), support (*n* = 47, 100%), and medical management (*n* = 47, 100%) were important (Figure 3). Almost all of the providers who responded agreed or strongly agreed that lactation education (*n* = 46, 97.8%) and support (*n* = 47, 100%) by an IBCLC would be beneficial for their patients (Figure 3).

**Figure 4:**
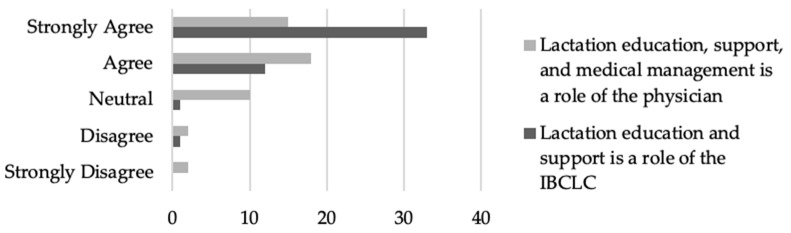
Provider beliefs about the role of the physician and IBCLC in providing lactation services to patients.

Of the medical providers that responded, almost all who responded (*n* = 45, 95.7%) believe that lactation education and support is the role of the IBCLC. Interestingly, of the providers who responded, 70.2% (*n* = 33) agreed or strongly agreed that lactation education, support, and medical management is the role of the physician (Figure 4).

**Figure 5:**
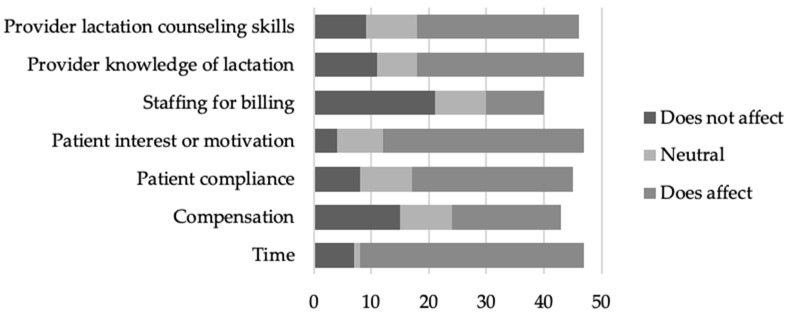
Provider reported barriers to providing lactation education and counseling to their patients.

Providers who responded indicated that time (*n* = 39, 83.0%), patient interest or motivation (*n* = 35, 74.5%), provider knowledge of lactation (*n* = 29, 61.7%), and provider lactation counseling skills (*n* = 28, 60.9%) were barriers to providing lactation education and counseling to their patients (Figure 5).

**Figure 6:**
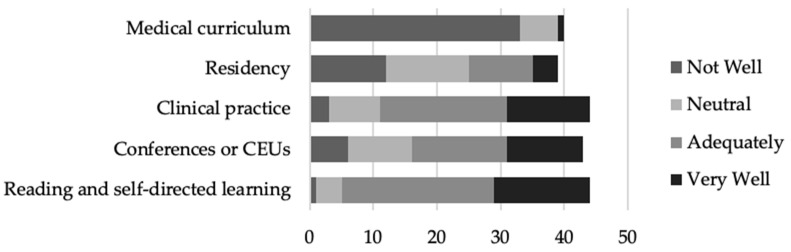
Provider beliefs about the extent to which they were provided with knowledge of lactation in different stages of their medical training.

Of the providers that responded, most felt that clinical practice (*n* = 33, 75.0%), conferences or CEUs (*n* = 27, 62.8%), and reading and self-directed learning prepared (*n* = 39, 88.6%) them “adequately” or “very well”. Conversely, the majority of providers that responded felt that their medical curriculum (*n* = 39, 97.5%) and residency (*n* = 25, 64.1%) either did not prepare them well or they felt neutral about the knowledge provided (Figure 6).

### 3.3. Provider Practices

The majority of the respondents providing care during pregnancy (*n* = 31) report initiating conversations about infant feeding ‘always’ (*n* = 9, 29.0%) or ‘most of the time’ (*n* = 10, 32.3%) during pregnancy (Table 3). Despite the frequency of initiating a conversation, only 22.6% (*n* = 8) report spending 11 min or more discussing breastfeeding during pregnancy (Table 3). The time discussing breastfeeding during the postpartum period (*n =* 68) does improve slightly, with 33.5% (*n* = 23) of respondents spending 11 min or more discussing breastfeeding (Table 3).

Providers report that 73.3%, 46.7%, 51.7%, and 42.7% of patients are being educated on lactation, formula feeding, mixed feeding, and complementary feeding during pregnancy, respectively (Table 3). This does not shift during the postpartum period, with providers reporting that 75.7%, 47.4%, 52.3%, and 44.4% of patients are being educated on lactation, formula feeding, mixed feeding, and complementary feeding, respectively (Table 3). Additionally, providers report that during the postpartum period, 57.4%, 40.8%, and 58.2% of patients are receiving lactation support, lactation medical management, and infant feeding support, respectively (Table 3).

After confirming the data met the assumptions of normality, linearity, homoscedasticity, and absence of multicollinearity, the researcher ran a multiple regression analysis to determine what factors, if any, predict patients receiving lactation education in the primary care setting. The full model includes the percentage of patients receiving lactation education as the dependent variable and provider age, whether or not the provider is an IBCLC, provider years of experience in maternal-child health, population density of the practice, the frequency that the provider initiates a conversation about breastfeeding during pregnancy, average provider preparedness and comfort with lactation support and medical management, and whether there is any lactation support available in their practice. The full model accounts for 81.8% of the variance (R^2^ = 0.818, F (7, 21) = 9.015, *p* < 0.001, CI = 0.728 to 0.910) in patients receiving lactation education in a primary care setting.

Although the entire model was significant, age (β = −0.636, t = −3.043, *p* = 0.009, CI = −3.03 to −0.525), years of experience in maternal-child health (β = 0.795, t = 3.761, *p* = 0.002, CI = 1.088 to 3.974), population density of the practice (β = 0.367, t = 2.833, *p* = 0.013, CI = 2.632 to 19.035), and average provider preparedness and comfort with lactation support and medical management (β = 0.427, t = 3.130, *p* = 0.007, CI = 4.035 to 21.606) most significantly contributed to this model when controlling for the other predictors (Table 4). For every percentage increase in lactation education, the provider age decreased by 1.78 years and the years of experience in maternal-child health went up by 2.53. Additionally, as the percentage of lactation education provided increased, so did the population density and average provider preparedness and comfort with lactation support and medical management.

### 3.4. Coordination of Care across Ancillary Providers

Approximately half (50.7%) of providers indicated that they had an IBCLC at their facility that was available to provide lactation support. The number of facilities with some level of feeding support on site increased to three quarters (72.5%) when credentials other than an IBCLC were considered as well, including CLC/CLEs, RDs, and CNMs. Despite infant feeding support on site being common in the practices of our respondents, referral frequency is highly variable both during pregnancy and postpartum. Most providers reported that they are referring to lactation professionals less than 40% of the time during pregnancy or postpartum (*n* = 8, 25.9% and *n* = 16, 23.6%, respectively; Table 5). Of the providers who give care during pregnancy (*n* = 31), the most frequently reported reasons for referrals to lactation professionals were maternal anxiety about breastfeeding (*n* = 19, 61.3%) and prenatal breastfeeding education (*n* = 16, 51.6%, Table 5). For providers giving care during the postpartum period for mother or baby (*n* = 68), the most frequently reported reasons for referrals to lactation professionals were difficulty latching (*n* = 40, 58.8%), maternal anxiety about breastfeeding (*n* = 37, 54.4%), and low milk supply (*n* = 34, 50.0%; Table 5).

## 4. Discussion

Improving breastfeeding exclusivity and duration has been shown to be positively influenced by both prenatal and postpartum breastfeeding support [6,7,8,9,10,12]. Additionally, previous research has established that educational interventions can alter provider practices related to lactation [14,15,16,17,18]. Through multiple regression analysis, these data have identified significant factors influencing whether patients receive lactation education in a primary care setting, including provider age, provider years of experience in maternal-child health, population density of the practice, and average provider preparedness and comfort with lactation support and medical management (Table 4). Additionally, participants echoed the importance of provider preparedness and comfort by indicating that provider lactation counseling skills and provider knowledge of lactation served as barriers to providing lactation education to their patients (Figure 5).

Provider preparedness and comfort with lactation support and medical management is a modifiable factor involved in predicting the frequency of lactation education. Unfortunately, many providers are reporting that they are not feeling appropriately prepared in either their medical curriculum or residency experiences (Figure 6). While incorporation of a robust lactation education into medical curriculums and/or residency training would be ideal, it is also possible that skilled lactation professionals can be utilized to fill the gaps. Respondents almost unanimously agreed that lactation support and medical management is the role of an IBCLC (Figure 4), and many felt that lactation education and support from an IBCLC would be beneficial to their patients (Figure 3). Despite this fact, less than 20% of respondents indicated that they are referring to lactation professionals 60% of the time or more (Table 5).

These data support a two-pronged approach to improving breastfeeding exclusivity and duration through primary care settings: improving primary care provider preparedness and comfort with lactation support and medical management and supporting coordination of care to skilled lactation providers. Educating providers will improve preparedness and comfort, both of which are necessary to ensuring that lactation is prioritized and that issues with lactation are identified early and accurately. Future research is needed to develop and pilot lactation curriculum tailored for medical students and residents working with maternal-child populations. For those providers already working in the community, programs providing additional education or guidelines for helping to protect and promote breastfeeding in primary care settings are needed.

Given that our data indicate that time is the number one barrier experienced by providers in delivering lactation education (Figure 5), coordination of care to IBCLCs and other skilled lactation professionals will be equally as important in ensuring that an appropriate level of support can be provided to dyads. Further research investigating models for coordination of care within the primary care setting or through collaboration with standalone outpatient and private practice lactation clinics is necessary to inform best practices. It must also be considered that the data gathered in this study have provided data specific to primary care settings in the Southeastern United States. Given that international healthcare systems may function differently, the survey would need to be repeated to develop models specific to each healthcare system. Additionally, given that this study reports the first distribution of the survey and that no other data of this kind are currently available for comparison, it will be important for the study to be repeated on a larger scale within the United States to ensure interventions are properly targeted.

## 5. Conclusions

A multiple regression analysis has identified average provider preparedness and comfort with lactation support and medical management as a modifiable predictor of patients receiving lactation education in the primary care setting. The percentage of patients receiving lactation education in the primary care setting may be increased with interventions aimed at improving primary care provider preparedness and comfort with lactation support and medical management. The effectiveness of such an intervention may also be improved with systems facilitating coordination of care to skilled lactation providers when necessary.

## Figures and Tables

**Table 1:** Demographic and clinical characteristics of respondents.

Characteristics	Values
**Age (*n* = 67; mean ± SD; years)**	39.7 ± 11.6
**Gender [*n* (%)]**	
Male	10 (14.5)
Female	59 (85.5)
**Credentials [*n* (%)]**	
MD/DO	41 (59.4)
NP	2 (2.9)
RN	10 (14.5)
RD	15 (21.7)
CNM	1 (1.4)
**IBCLC Certification [*n* (%)]**	
Yes	14 (20.3)
No	55 (79.7)
**Practice Specialty [*n* (%)] ^a^**	
Obstetrics and Gynecology	9 (13)
Pediatrics	35 (50.7)
Family Medicine	3 (4.3)
Maternal-child Health (MCH)	5 (7.2)
Other	7 (10.1)
**Years in MCH Practice (mean ± SD; years)**	11.8 ± 11.7
**Practice Setting [*n* (%)]**	
Outpatient	26 (37.7)
Inpatient and Outpatient	43 (62.3)
**Practice Affiliation [*n* (%)]**	
Private Healthcare System	12 (17.4)
Public/Academic Healthcare System	34 (49.3)
Public Health Clinic	6 (8.7)
Private Practice	14 (20.3)
Other	3 (4.3)
**Population Served [*n* (%)] ^b^**	
Pregnant	31 (44.9)
Postpartum	35 (50.7)
Infants	55 (79.7)
**Geographic Setting [*n* (%)] ^a^**	
Urban (>50,000)	37 (53.6)
Suburban	16 (23.2)
Small City	14 (20.3)
Rural (<2500)	1 (1.4)

^a^ Characteristics do not sum to total due to missing data. ^b^ Total sums to more than 100% due to ability to “select all that apply”.

**Table 2:** Frequency of providers receiving requests for information during pregnancy and postpartum by topic.

Topic	Providers Receiving Requests during Pregnancy [*n* (%)] ^a^	Providers Receiving Requests Postpartum [*n* (%)] ^a^
Breastfeeding	25 (80.6)	39 (57.4)
Formula Feeding	16 (51.6)	26 (38.2)
Mixed Feeding	13 (41.9)	24 (35.3)
Complementary Feeding	9 (29.0)	14 (20.6)
Nutrition for Lactation	13 (41.9)	19 (27.9)
Milk/Drug Interactions	9 (29.0)	14 (20.6)
Caffeine Consumption during Lactation	9 (29.0)	11 (16.2)
Alcohol Consumption during Lactation	10 (32.3)	13 (19.1)
Other	1 (3.2)	4 (5.9)

^a^ Total sums to more than 100% due to ability to “select all that apply”.

**Table 3:** Provider practices related to infant feeding during pregnancy and postpartum.

Provider Practice	Pregnancy	Postpartum
**Initiating a Conversation about Breastfeeding [*n* (%)] ^a^**		
Never	0 (0)	N/A
Sometimes	2 (6.5)	N/A
Half of the time	4 (12.9)	N/A
Most of the time	10 (32.3)	N/A
Always	9 (29.0)	N/A
**Time Discussing Breastfeeding [*n* (%)] ^a^**		
Not Applicable	0 (0)	2 (2.9)
<5 min	9 (29.0)	10 (14.7)
5–10 min	9 (29.0)	12 (17.6)
11–15 min	2 (6.5)	10 (14.7)
16–30 min	1 (3.2)	3 (4.4)
30+ min	4 (12.9)	10 (14.7)
**Provider Reported Percentage of Patients Supported [% ± SD]**		
Lactation education	73.3 ± 28.8	75.7 ± 26.9
Formula feeding education	46.7 ± 28.8	47.4 ± 29.2
Mixed feeding education	51.7 ± 29.5	52.3 ± 30.2
Complementary feeding education	42.7 ± 35.7	44.4 ± 35.8
Lactation support	N/A	57.4 ± 36.4
Lactation medical management	N/A	40.8 ± 35.2
Infant feeding support (mixed feeding, formula feeding, bottles)	N/A	58.2 ± 32.6

^a^ Characteristics do not sum to total due to missing data. N/A = not applicable as question was not included in the survey.

**Table 4:** Multiple regression results and significance testing.

	Full Model
*N*	22
R^2^	0.818
F (7, 21)	9.015 **
β	
Provider age	−0.636 *
Whether or not the provider is an IBCLC	−0.234
Provider years of experience in MCH	0.795 **
Population density of the practice	0.367 *
Frequency provider initiatives a conversation about breastfeeding during pregnancy	0.260
Average provider preparedness and comfort with lactation support and medical management	0.427 *
Whether or not lactation support is available in the practice	0.227

Note: * *p* < 0.05; ** *p* < 0.005.

**Table 5:** Provider referral practices related to infant feeding during pregnancy and postpartum.

Provider Practice	Pregnancy	Postpartum
**Frequency of Referrals to Lactation Professionals [*n* (%)] ^a^**		
0–20%	6 (19.4)	11 (16.2)
21–40%	2 (6.5)	5 (7.4)
41–60%	5 (16.1)	4 (5.9)
61–80%	1 (3.2)	4 (5.9)
81–100%	5 (16.1)	8 (11.7)
**Reason for Referral to Lactation Professional [*n* (%)] ^b^**		
Insufficient weight gain	N/A	30 (44.1)
Difficulty latching	N/A	40 (58.8)
Maternal anxiety about breastfeeding	19 (61.3)	37 (54.4)
Low milk supply	N/A	34 (50.0)
Prenatal breastfeeding education	16 (51.6)	19 (27.9)
Chronic plugged ducts	N/A	18 (26.5)
First time breastfeeding	1 (3.2)	1 (1.5)

^a^ Characteristics do not sum to total due to missing data. ^b^ Total sums to more than 100% due to ability to “select all that apply”. N/A = not applicable as question was not included in the survey.

## Data Availability

Data are contained within the article.

## Appendix A

**Table A1:** Identified domains, example sub-dimensions, and question examples resulting from the initial comprehensive literature review of lactation practices in primary care settings.

Identified Domain	Example Sub-Dimensions	Question Examples
Provider Perceptions and Roles	Provider perceptions of patient interest in lactation Provider willingness to provide lactation support, provider readiness to provide lactation management Barriers to lactation education in primary care settings	How often do your patients initiate a conversation about infant feeding during pregnancy? What infant feeding topics do your pregnant and lactating clients request information about? What percentage of your clients are receiving information about breastfeeding during your encounter?
Provider Practices	Processes for delivery of lactation education Time spent providing lactation education and support Perception of knowledge related to lactation support and medical management	How long do you spend per encounter, on average, discussing breastfeeding with your patients? Do you consider lactation education important to provide for patients during pregnancy?
Coordination of Care Across Ancillary Providers	Provider perceptions of roles associated with lactation support Processes for referral to ancillary providers Frequency of referral to ancillary providers	How many of your patients do you refer to lactation professionals? In what cases do you refer your patients to a lactation professional?

**Table A2:** Definitions for lactation education, lactation support, and lactation medical management utilized to gather data related to practices in primary care settings.

Term	Definition
Lactation Education	Any intervention aimed at increasing knowledge and skills related to the delivery of human milk to infants. Examples include, but are not limited to, information about frequency of infant feeding, infant behavior surrounding feeding, and guiding parents on how to know their baby is getting enough milk.
Lactation Support	Counseling, encouragement, and management of clinical challenges related to human milk feeding. Examples include, but are not limited to, latch, positioning, and appropriate stimulation of the breasts when not directly breastfeeding.
Lactation Medical Management	Management of any condition requiring clinical diagnosis and treatment by an appropriately licensed healthcare provider. Examples include, but are not limited to, mastitis, postpartum depression, pharmaceutical interventions for increasing milk supply, and tongue-tie diagnosis.
